# Molecular basis for the binding and modulation of V-ATPase by a bacterial effector protein

**DOI:** 10.1371/journal.ppat.1006394

**Published:** 2017-06-01

**Authors:** Jianhua Zhao, Ksenia Beyrakhova, Yao Liu, Claudia P. Alvarez, Stephanie A. Bueler, Li Xu, Caishuang Xu, Michal T. Boniecki, Voula Kanelis, Zhao-Qing Luo, Miroslaw Cygler, John L. Rubinstein

**Affiliations:** 1 The Hospital for Sick Children Research Institute, Toronto, Ontario, Canada; 2 Department of Medical Biophysics, University of Toronto, Toronto, Ontario, Canada; 3 Department of Biochemistry, University of Saskatchewan, Saskatoon, Saskatchewan, Canada; 4 Department of Biological Sciences, Purdue University, West Lafayette, Indiana, United States of America; 5 Department of Chemistry, University of Toronto, Toronto, Ontario, Canada; 6 Department of Chemical and Physical Sciences, University of Toronto Mississauga, Mississauga, Ontario, Canada; 7 Department of Cell and Systems Biology, University of Toronto, Toronto, Ontario, Canada; 8 Department of Biochemistry, University of Toronto, Toronto, Ontario, Canada; Gifu University, JAPAN

## Abstract

Intracellular pathogenic bacteria evade the immune response by replicating within host cells. *Legionella pneumophila*, the causative agent of Legionnaires’ Disease, makes use of numerous effector proteins to construct a niche supportive of its replication within phagocytic cells. The *L*. *pneumophila* effector SidK was identified in a screen for proteins that reduce the activity of the proton pumping vacuolar-type ATPases (V-ATPases) when expressed in the yeast *Saccharomyces cerevisae*. SidK is secreted by *L*. *pneumophila* in the early stages of infection and by binding to and inhibiting the V-ATPase, SidK reduces phagosomal acidification and promotes survival of the bacterium inside macrophages. We determined crystal structures of the N-terminal region of SidK at 2.3 Å resolution and used single particle electron cryomicroscopy (cryo-EM) to determine structures of V-ATPase:SidK complexes at ~6.8 Å resolution. SidK is a flexible and elongated protein composed of an α-helical region that interacts with subunit A of the V-ATPase and a second region of unknown function that is flexibly-tethered to the first. SidK binds V-ATPase strongly by interacting via two α-helical bundles at its N terminus with subunit A. *In vitro* activity assays show that SidK does not inhibit the V-ATPase completely, but reduces its activity by ~40%, consistent with the partial V-ATPase deficiency phenotype its expression causes in yeast. The cryo-EM analysis shows that SidK reduces the flexibility of the A-subunit that is in the ‘open’ conformation. Fluorescence experiments indicate that SidK binding decreases the affinity of V-ATPase for a fluorescent analogue of ATP. Together, these results reveal the structural basis for the fine-tuning of V-ATPase activity by SidK.

## Author summary

V-ATPase-driven acidification of lysosomes in phagocytic cells activates enzymes important for killing of phagocytized pathogens. Successful pathogens can subvert host defenses by secreting effectors that target V-ATPases to inhibit lysosomal acidification or lysosomal fusion with other cell compartments. This study reveals the structure of the V-ATPase:SidK complex, an assembly formed from the interaction of host and pathogen proteins involved in the infection of phagocytic white blood cells by *Legionella pneumophila*. The structure and activity of the V-ATPase is altered upon SidK binding, providing insight into the infection strategy used by *L*. *pneumophila* and possibly other intravacuolar pathogens.

## Introduction

Acidification of intracellular compartments by vacuolar-type ATPases (V-ATPases) is crucial for numerous biological processes [1,2]. These processes include glycosylation in the Golgi [3,4], loading of neurotransmitters in secretory vesicles [5,6], protein trafficking in endosomes [7–9], and amino acid sensing in lysosomes [10,11]. V-ATPases pump protons across a phospholipid membrane using energy from the hydrolysis of adenosine triphosphate (ATP) to adenosine diphosphate (ADP) and inorganic phosphate [1,12]. In the yeast *Saccharomyces cerevisiae* the complex is composed of subunits A_3_B_3_CDE_3_FG_3_Hac_8_c′c″def [13], where subunits denoted by upper case letters form the soluble catalytic V_1_ region while subunits denoted by lower case letters form the membrane-embedded V_O_ region. ATP hydrolysis occurs in the V_1_ region, where three catalytic heterodimers of A- and B-subunits assemble into a pseudo-symmetric trimer of AB heterodimers [14–16]. Each AB heterodimer contains a catalytic nucleotide-binding site and each is found in a different conformation [17] termed ‘tight’, ‘loose’, and ‘open’ with bound ATP, bound ADP and phosphate, and no nucleotide expected to be bound, respectively. Conformational changes in the AB heterodimers are coupled to proton translocation across the V_O_ region by a rotary catalytic mechanism [18,19] where the rotor subcomplex, consisting of subunits DFc_8_c′c″d, turns relative to the rest of the complex. Under certain conditions, the V_1_ region can dissociate from the V_O_ region to inhibit ATP hydrolysis [20–23] and prevent proton translocation. Aside from dissociation of the complex, little is known about how the activity of V-ATPases is regulated.

V-ATPase activity has a central role in the clearance of material phagocytosed by immune cells. Killing of pathogens by phagocytic white blood cells, such as macrophages, occurs in phagolysosomes [24], which are acidified by V-ATPases [25]. This acidification leads to the activation of enzymes that help to destroy phagocytized material. Some intracellular bacteria subvert this process by secreting effectors that inhibit either the formation or acidification of phagolysosomes [25–27]. The protein SidK, secreted by *Legionella pneumophila* [28,29], interacts with the V-ATPase to inhibit acidification of the phagolysosome in the early stages of infection [25]. However, the molecular basis of this interaction and inhibition remain unclear. In this study, we determined the structure of the N-terminal domain of SidK at 2.3 Å resolution by X-ray crystallography, revealing SidK to be a flexible and elongated protein. Electron cryomicroscopy (cryo-EM) has recently emerged as a powerful method to analyse the structure of the V-ATPase at subnanometer resolution [13,30,31]. Although SidK normally binds to the human V-ATPase, to date it has only been possible to perform sub-nanometer resolution cryo-EM with V-ATPase from *S*. *cerevisiae* [30] or the insect *Manducca sexta* [31] due to abundance of the enzyme. Therefore, we used the *S*. *cerevisiae* V-ATPase to determine the structure of a V-ATPase:SidK_3_ complex at 6.8 Å resolution by single particle cryo-EM, showing that SidK binds the N-terminal region of the V-ATPase A-subunit. SidK binding to the V-ATPase inhibits V-ATPase activity by ~40%. Consistent with this subtle fine-tuning of V-ATPase activity, the structures do not reveal significant conformation rearrangement on binding. Instead, SidK binding reduces A-subunit flexibility and decreases the affinity of V-ATPase for the fluorescent ATP analogue TNP-ATP. The full neutralization of the *Legionella* containing vacuole (LCV) in the early stages of infection [32] therefore likely involves other effectors and the extended C-terminal domain of SidK seen in our model suggests that there are additional roles for this part of the protein.

## Results

### Crystal structures of SidK

**Fig 1:**
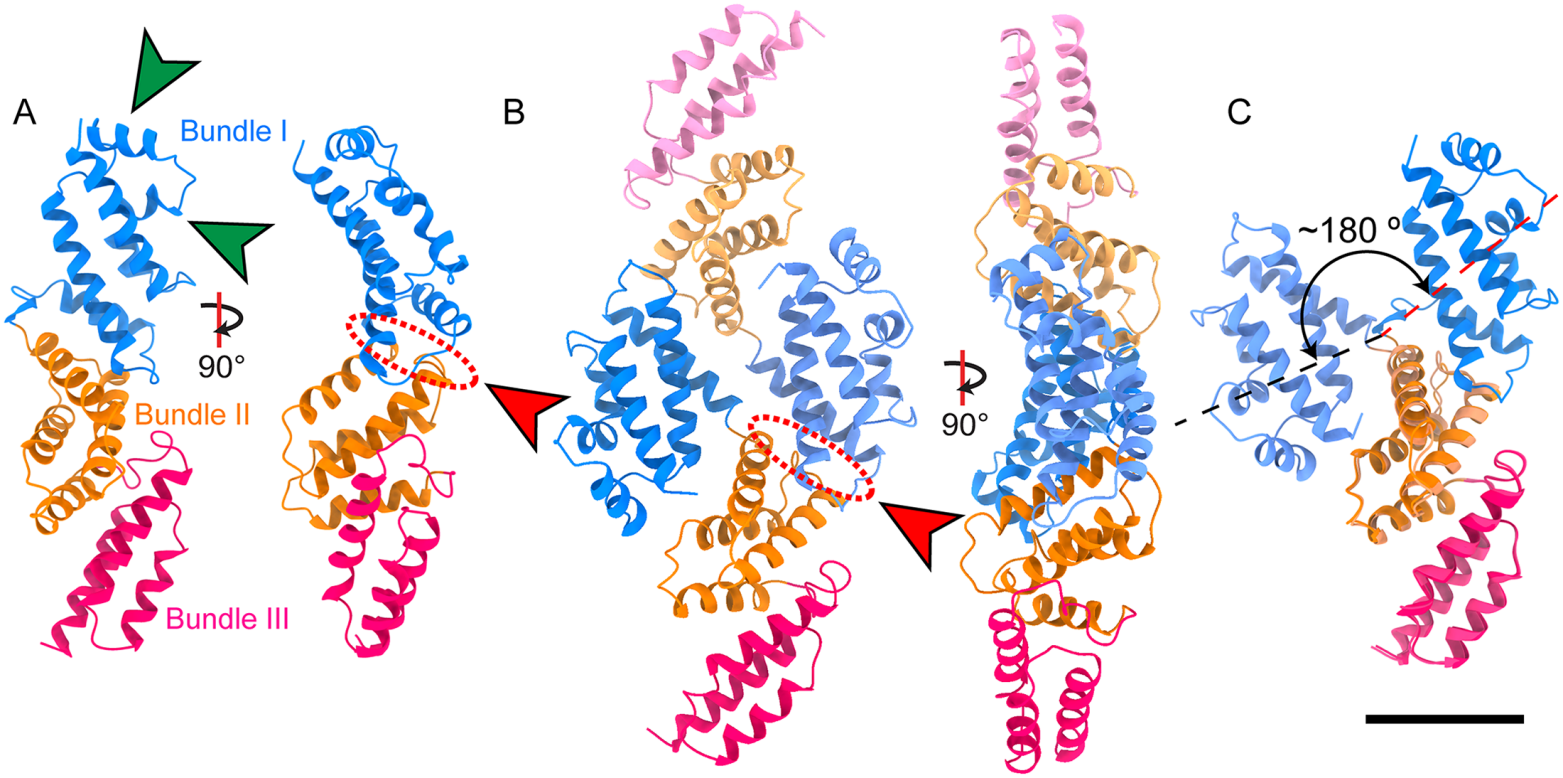
Crystal structures of SidK N-terminal domain. Structures of SidK(16–278) were determined where one (**A**) and two (**B**) monomers were in the asymmetric unit. SidK(16–278) is an elongated protein comprised of three α-helical bundles ('Bundle I', 'Bundle II', 'Bundle III'). Bundle I includes two short α-helices that deviate from the main α-helical bundle axis (A, green arrowheads). **C**, In the crystal structure with two SidK monomers in the asymmetric unit, Bundle I is rotated ~180° with respect to Bundle II. This structural rearrangement allows the interface between Bundle I and Bundle II in the monomer structure (A, red oval and arrowhead) to be maintained in the dimer structure (B, red oval and arrowhead) through domain swapping between the two monomers. Scale bar: 25 Å.

In order to gain insight into the role of SidK in the intracellular survival of *L*. *pneumophila*, intact SidK (575 residues) and different truncations of the protein were expressed heterologously in *Escherichia coli*, purified, and crystallization trials were performed. One SidK construct, consisting of residues 16–278 (SidK-N), yielded two crystal forms that diffracted X-rays to 2.3 to 2.4 Å resolution and the structure of SidK-N was determined from these crystals. The first crystal form contained one molecule of SidK(16–278) in the asymmetric unit and the final model included amino acids 16–274. The protein has an elongated and slightly bent α-helical structure consisting of three α-helical bundles (Fig 1A). The three α-helical bundles contain four, four, and three α-helices, respectively, and are connected by loops in the bundles. The first α-helical bundle (α1–2, α5–6) also has an extension nearly perpendicular to the bundle axis that consists of two additional short α-helices (α3-α4) (Fig 1A, green arrowheads). Bioinformatic analysis of the SidK structure showed that the arrangements of α-helices in the second and third α-helical bundles are similar to the arrangement of α-helices observed in many other proteins, including DOCK2 (PDB ID 3A98) and endo-α-N-acetylgalactoseaminidase [33]. However, the N-terminal bundle with its extensions, which from previous analysis was proposed to be the region that interacts with the V-ATPase [25], does not resemble any other known protein structures. The second crystal form of SidK(16–278) contained two molecules in the asymmetric unit related by a non-crystallographic two-fold symmetry (Fig 1B). In this crystal form SidK-N exists as a dimer, with the two molecules swapping their second and third α-helical bundles (Fig 1A and 1B, red arrowheads and oval). This domain swapping is accomplished by a rotation of ~180° relative to the first α-helical bundle (Fig 1C) that occurs through conformational changes within the short linker connecting the first and second α-helical bundles (residues 117–125). The domain swapping retains the interface between the first and second α-helical bundles seen in the first crystal form and the domain-swapped monomer is similar to the monomer in the first crystal form (Fig 1A and 1B). The monomeric SidK predominates in solution, even at high concentrations, and therefore the elongated monomer structure is expected to be the functional state of the protein. Only the N-terminal region of SidK interacts with the V-ATPase and consequently a highly-elongated SidK structure was unexpected. However, the structure on its own did not provide clues into how SidK interacts with or inhibits the V-ATPase.

### SidK binds the N-terminal region of V-ATPase subunit A

**Fig 2:**
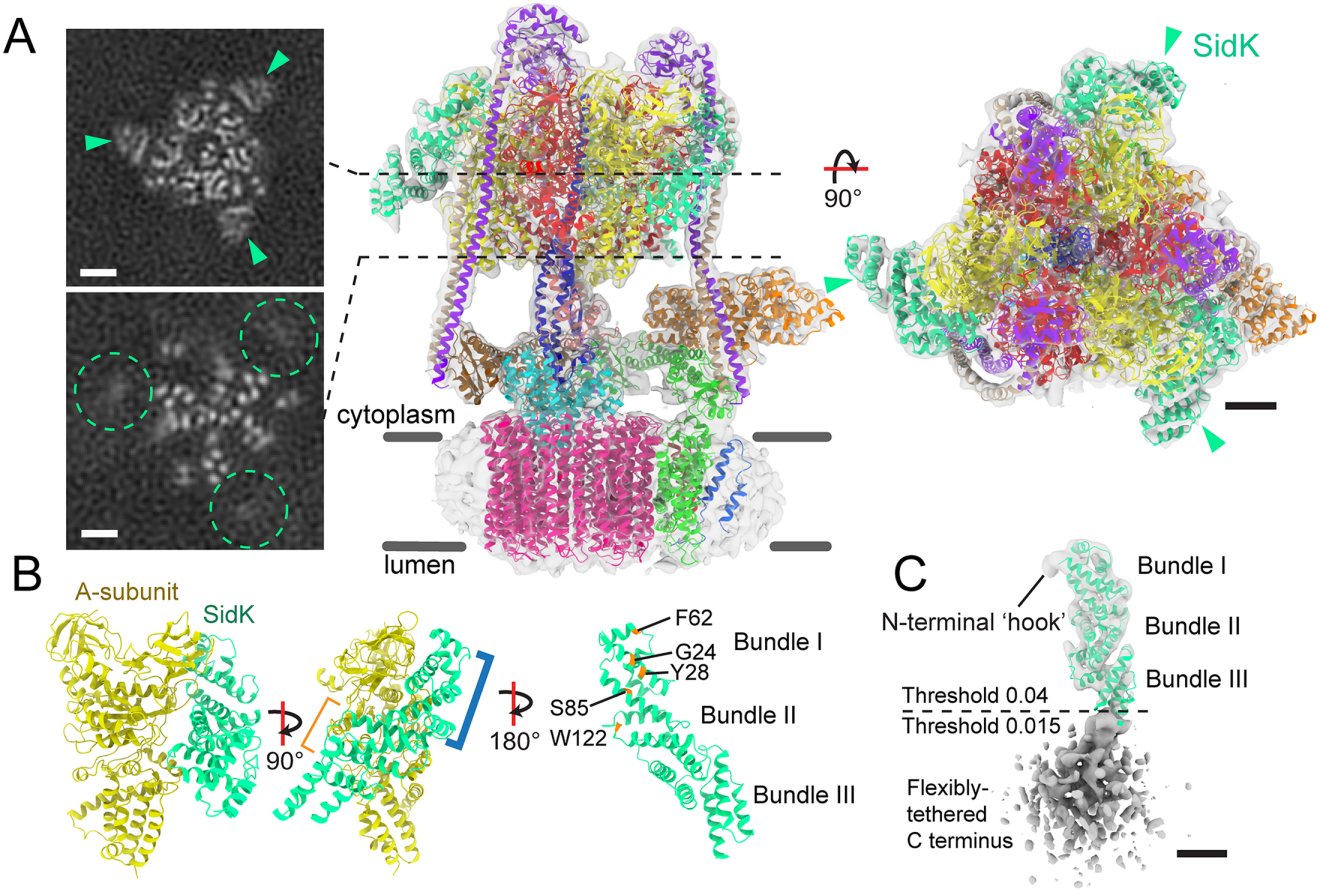
Cryo-EM structure of the V-ATPase:SidK_3_ complex. **A**, Three SidK proteins (*teal*) are bound to the V-ATPase in the soluble catalytic V_1_ region. The SidK density is well-defined in the N-terminal region where it binds the V-ATPase (teal arrowheads) and is poorly-resolved in the C-terminal region closer to the membrane-embedded part of the complex (teal dashed circles). **B**, SidK binds to the N-terminal region of subunit A of the V-ATPase. The major interaction surface is on SidK α-helical bundle I (blue bracket) and appears to involve residues G24, Y28, F62, S85, and W122. **C**, The cryo-EM map density (gray surface) reveals a feature resembling a 'hook' at the N terminus of SidK that is missing from the crystal structure. The C-terminal region of SidK is flexibly-tethered to its N-terminal region and is poorly resolved in the cryo-EM density. For clarity, the surface representation of SidK was rendered at a higher density threshold for the N-terminal domain (Threshold 0.04) than for the C-terminal domain (Threshold 0.015). Scale bars: 25 Å.

In order to understand how SidK interacts with the V-ATPase, purified full length SidK (residues 1 to 573) and detergent solubilized and purified intact V-ATPase from *S*. *cerevisiae* [23] were incubated together and the resulting complex analysed by cryo-EM (S1 Fig). In the absence of ATP, V-ATPase adopts three distinct conformations that correspond to different rotational positions of its rotor subcomplex relative to the rest of the enzyme [30]. Particle images were subjected to 3-D classification in order to separate these different conformations of the V-ATPase, as well as different occupancies of SidK binding to the V-ATPase (S2 Fig). The cryo-EM maps showed additional density corresponding to SidK attached to the A-subunits of the V-ATPase (Fig 2A, green arrowheads). While V-ATPase particles decorated with SidK were found in all three rotational states of the enzyme, the highest-quality cryo-EM map was obtained for V-ATPase in rotational state 1 fully decorated with three copies of SidK (Fig 2A and S2 Fig) and reached a resolution of 6.8 Å (S3 and S4 Figs). The C-terminal region of SidK had a local resolution worse than the rest of the complex (S4A Fig, 'C-term'). The distribution of particles in the different rotational states differed slightly from the distribution observed in the absence of SidK (S4B Fig): 49%, 23%, and 28% for rotational states 1, 2 and 3 with SidK bound versus 47%, 36%, and 17% without SidK bound [30]. A pseudo atomic model of the V-ATPase:SidK_3_ assembly was constructed using the cryo-EM map to guide flexible fitting of the crystal structure of SidK determined here, an earlier model of V-ATPase subunits A_3_B_3_CDE_3_FG_3_ [30], and a model of the V_O_ region subunits ac_8_c′c″def [13] (Fig 2A). Fitting of SidK into each of the three corresponding densities in the map required flexing the crystal structure only at the interface between α-helical bundles I and II. The flexibility between the first and second α-helical bundles is consistent with the flexibility in the linker between these bundles observed by X-ray crystallography. Bound to the V-ATPase, the C terminus of SidK extends toward the expected position of the membrane (Fig 2A). The flexing of the SidK model required to fit it into the cryo-EM map introduced a slight bend in the linker region around residues Lys123-Ser124. The fitting shows that SidK binds the V-ATPase A-subunit primarily via its first α-helical bundle (Fig 2B, blue bracket) and the three copies of SidK each interact mostly with the corresponding N-terminal region of one of the three A-subunits [25]. Mapping the contact surfaces in all three SidK:A-subunit pairs in the cryo-EM map to the SidK crystal structure, the main contact surface appears to involve residues Gly24, Tyr28, Phe62, Ser85, and Trp122 from the first α-helical bundle of SidK. Although the SidK crystal structure spans almost the entire region that binds the V-ATPase, it is missing the first 16 residues of the protein, which the cryo-EM map shows to form a hook-like feature that penetrates the non-catalytic interfaces between AB heterodimers (Fig 2C, 'hook'). The C-terminal region of SidK is poorly resolved in the map and has lower density than SidK-N, suggesting flexible tethering between the N- and C-terminal regions (Fig 2A and 2C, grey density). Surprisingly, comparison of the V-ATPase:SidK_3_ and V-ATPase [30] structures revealed no major conformational rearrangements in the V-ATPase upon SidK binding (S4C–S4E Fig). Furthermore, purification and structural analysis of substoichiometric V-ATPase:SidK assemblies (S2C Fig) did not show major conformational differences in the V-ATPase with substoichiometric SidK, compared to V-ATPase alone and V-ATPase that is fully-decorated by SidK.

### SidK is a specific partial inhibitor of purified V-ATPase

**Fig 3:**
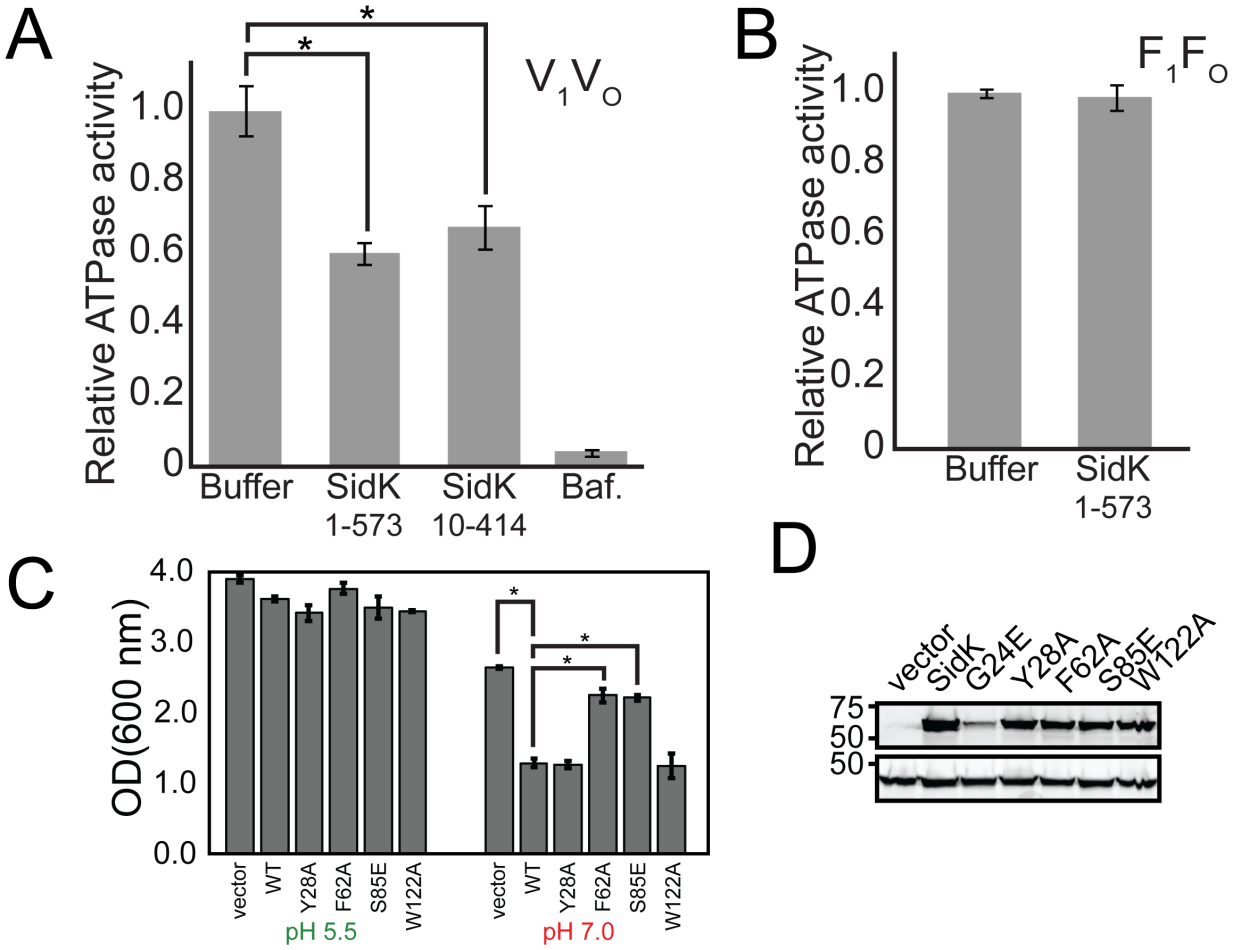
Inhibition of the V-ATPase by SidK. **A**, SidK, either full length or residues 10–414, inhibits V-ATPase by approximately 40% with 10× excess of SidK to available binding sites. “Baf.” indicates bafilomycin. **B**, SidK does not inhibit ATPase activity of the F-type ATP synthase, showing that it is a specific inhibitor of V-ATPases. **C**, Yeast expressing SidK and SidK point mutations show normal growth on medium at pH 5.5. Wild type SidK reduces yeast growth on medium buffered to pH 7.0, indicating V-ATPase inhibition. SidK point mutations F62A and S85E alleviate SidK inhibition of V-ATPase while point mutations Y28A and W122A do not. **D**, Western blotting shows that all of the point mutations tested are expressed in *S*. *cerevisae* at levels similar to wild type (PGK, phosphoglycerate kinase as a loading control), except G24E, which was consequently excluded from the analysis. *, p<0.01.

The main interaction between SidK and V-ATPase involves the ‘non-homologous regions’ of the A-subunits (Fig 2B). These regions are found in the catalytic A-subunits of V-ATPases, but not in the corresponding catalytic β-subunits of ATP synthases, suggesting that SidK inhibition is V-ATPase-specific. To test this hypothesis we performed *in vitro* ATPase inhibition assays with V-ATPase and the F-type ATP synthase. These assays showed that SidK inhibits V-ATPase by ~40% (Fig 3A) but does not detectably inhibit the F-type ATP synthase (Fig 3B). Partial inhibition of the V-ATPase by SidK is not surprising, as complete inhibition of V-ATPase activity is well known to kill mammalian cells [34], which would not be advantageous for *L*. *pneumophila* infection. A construct consisting of residues 10 to 414 could inhibit V-ATPase to the same extent as the full length SidK (Fig 3A), showing that the N-terminal ‘hook’ feature seen in Fig 2C is not essential for inhibition by SidK. SidK was first identified as a V-ATPase binding protein because its expression in yeast reduced growth in liquid medium buffered to pH 7.0 [25], a characteristic of V-ATPase deficiency. We found that yeast expressing SidK from a plasmid were still able to grow on solid rich medium supplemented with 4 mM ZnCl_2_, indicating residual V-ATPase activity and consistent with the partial inhibition seen in *in vitro* assays (Fig 3A). Residues from the first α-helical bundle of SidK that appear to interact with V-ATPase (Fig 2B, Gly24, Tyr28, Phe62, Ser85, and Trp122) are highly conserved in SidK orthologs from other *Legionella* species, with Ser85 substituted to Ala in some distant *Legionella* species [35]. α-helical bundles II and III of SidK had almost no exposed conserved residues, except for Glu220. In order to test the importance of SidK residues as well as the model for V-ATPase binding presented above, SidK point mutations G24E, Y28A, F62A, S85E, and W122A were generated in both yeast and bacterial expression vectors. Using the bacterial expression vectors, mutant SidK was purified and all of the constructs were found to have melting temperatures within 0.5°C of the 54.1°C Tm found for wild type SidK. Mutant SidK constructs in yeast expression vectors were used to transform yeast, and yeast growth was monitored at pH 5.5 and 7.0, the latter requiring fully functional V-ATPase for optimal growth. This assay showed that the point mutations F62A and S85E are sufficient to prevent SidK from inhibiting V-ATPase and allow normal yeast growth (Fig 3C and 3D). Mutants Y28A and W122A were indistinguishable from wild type SidK while G24E expressed at a lower level than the other SidK constructs and consequently its effect on V-ATPase activity could not be characterized (Fig 3D). The SidK mutants F62A and S85E (S5A Fig). However, unlike wild type SidK (S1A Fig), could not be co-purified with V-ATPase, suggesting significantly decreased affinity for the V-ATPase. The F62A and S85E mutations also prevent SidK from inhibiting ATPase activity of the V-ATPase *in vitro* (S5B Fig). For these ATPase assays, which required large quantities of purified V-ATPase and simultaneous availability of wild type SidK and mutant SidK at identical concentration, SidK samples were frozen before use. Possibly as a result of this freezing, wild type SidK only inhibited V-ATPase by ~30%, not the ~40% inhibition seen with freshly purified wild type SidK (Fig 3A). The ability of the F62A and S85E mutations to prevent SidK from inhibiting V-ATPase supports the proposed model of SidK:V-ATPase complex formation.

### SidK binding decreases ‘open’ conformation mobility

**Fig 4:**
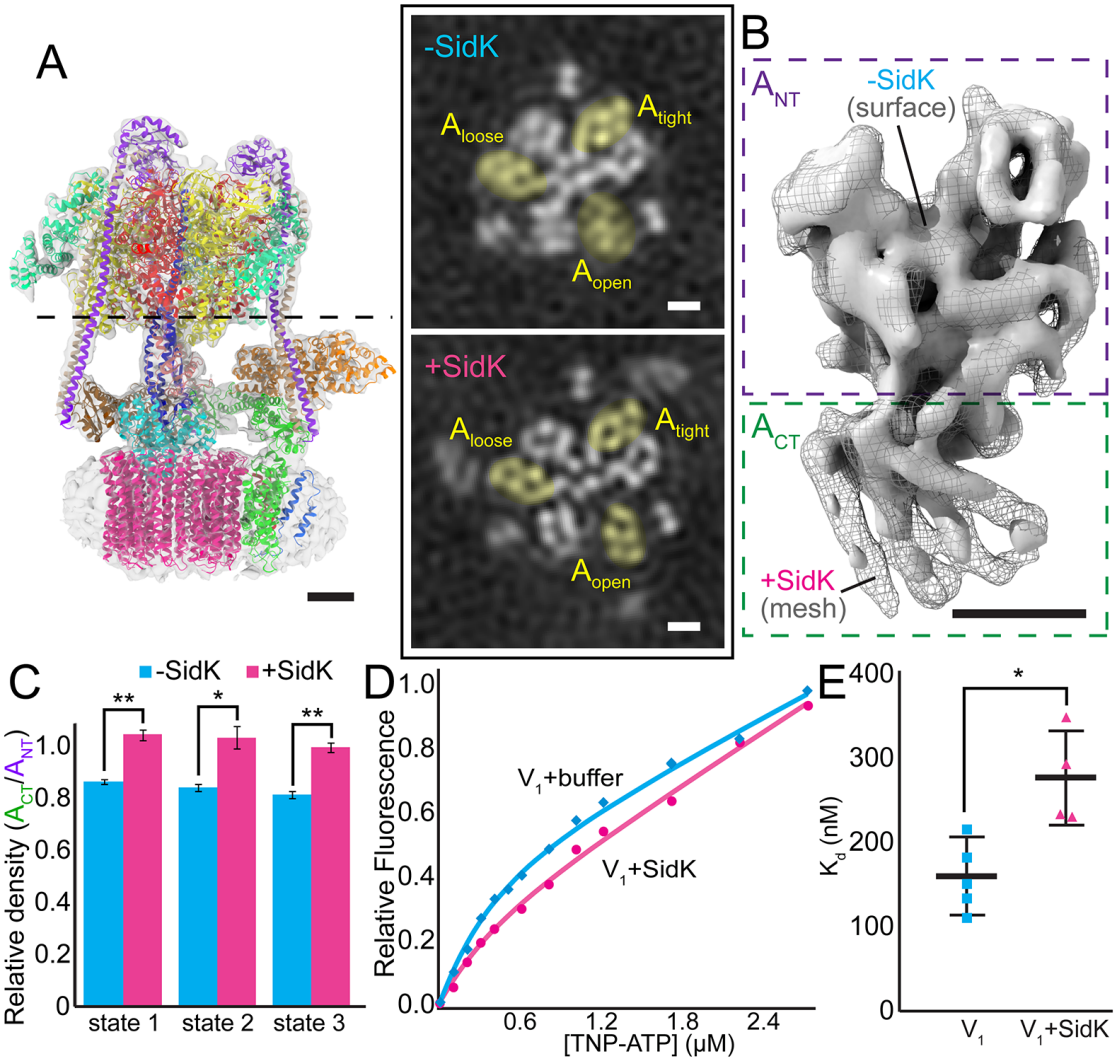
SidK reduces the flexibility of the A-subunit and its binding affinity for TNP-ATP. **A**, Slices through the cryo-EM maps of V-ATPase and V-ATPase:SidK_3_ in state 3, low-pass filtered to 10 Å. In V-ATPase without SidK (-SidK), the C-terminal region of the A-subunit (A_CT_) in the ‘open’ conformation (A_open_) has lower density compared to the A-subunit in the ‘tight’ or ‘loose’ conformations (A_tight_, A_loose_). With SidK bound (+SidK), density for the ‘open’ A-subunit is comparable to other subunits in the V-ATPase:SidK_3_ map. **B**, Comparison between the A-subunits in the ‘open’ conformation from V-ATPase (*gray surface*) and V-ATPase:SidK_3_ (*gray mesh*) cryo-EM maps. A_CT_ is better defined with SidK bound. Scale bars: 25 Å. **C**, The average density of A_CT_ relative to A_NT_ is higher with SidK bound to V-ATPase in all rotational states. **D**, Representative relative fluorescence values and fitted curves from titration of TNP-ATP into a solution containing V_1_ alone (*blue*) or V_1_ with SidK (*pink*). **E**, Dissociation constant K_d_ values calculated from data in (D) show increased K_d_ values with SidK treatment, indicating that SidK binding decreases the affinity of the V_1_ subcomplex for TNP-ATP. *, p<0.01; **, p<0.001.

As described, the V-ATPase:SidK_3_ structure by itself did not offer an explanation for the ~40% inhibition of V-ATPase activity that occurs on SidK binding. However, comparison of a map of V-ATPase alone [30] with the V-ATPase:SidK_3_ structure showed a subtle difference. In the earlier cryo-EM maps of the V-ATPase alone, the C-terminal region of the A-subunit in the ‘open’ conformation had a lower density relative to A-subunits in the ‘tight’ or ‘loose’ conformations (Fig 4A upper, yellow densities). The cryo-EM map of V-ATPase alone was determined with identical specimen preparation, imaging, and image processing conditions. The decreased density indicates mobility of this protein domain. In comparison, the C-terminal region of the ‘open’ A-subunit in the V-ATPase:SidK_3_ cryo-EM map is well defined (Fig 4A lower, yellow densities, and 4B, surface versus mesh), indicating a more rigid structure when SidK is bound. This higher density for the ‘open’ A-subunit with SidK bound compared to the ‘open’ A-subunit without SidK is seen in all three rotational states of the enzyme (Fig 4C). Furthermore, subtraction of the V-ATPase map from the V-ATPase:SidK_3_ map, although dominated by the presence of SidK, shows residual density in all three rotational states that is consistent with a more rigid A-subunit with SidK bound (S6 Fig). Estimation of the local resolution of the density maps also shows higher relative resolution estimates for the ‘open’ A-subunit C-terminal region in V-ATPase:SidK_3_ than in V-ATPase alone (S4A Fig), further suggesting reduced mobility upon SidK binding.

### SidK reduces the affinity of the V_1_ region for ATP

The ‘open’ A-subunit is poised to bind nucleotide and flexibility is essential for rotary catalysis in the V-ATPase and other rotary ATPases [30,36]. The binding of SidK to the A-subunit and the effect of SidK on the flexibility of this subunit suggested that SidK may alter binding of nucleotide to the AB heterodimers. The effect of SidK on the affinity of V-ATPase for ATP was investigated using the soluble V_1_ subcomplex purified from *S*. *cerevisiae*. As expected, the purified V_1_ subcomplex had no detectable ATPase activity [22]. However, the V_1_ subcomplex was still able to bind nucleotide as well as SidK (Fig 4D and S1A Fig), suggesting that the catalytic A_3_B_3_ hexamer of the V_1_ subcomplex remains in a conformation similar to the intact V-ATPase assembly and that the auto-inhibition of the V_1_ region does not interfere with accessibility of the active site. The binding affinity of nucleotide to the V_1_ subcomplex was determined using the fluorescent TNP-ATP. Although the affinities of a protein for ATP and TNP-ATP may be different [37], this fluorescent analogue of ATP has been used successfully to compare changes in nucleotide binding in a variety of experiments [37–39]. TNP-ATP was titrated into a solution containing the purified V_1_ assembly and fluorescence was measured (Fig 4D). The increase in fluorescence was modeled to calculate the equilibrium dissociation constant K_d_ (Fig 4D) according to Eqs 1 and 2 (see Methods). The calculated K_d_ of V_1_:TNP-ATP (160 +/- 50 nM) was approximately two-fold lower than the K_d_ of V_1_:SidK:TNP-ATP (280 +/- 60 nM), indicating that SidK decreases the affinity of the V-ATPase soluble catalytic region for TNP-ATP. This decrease in nucleotide affinity is consistent with the decreased flexibility of the A-subunit of V-ATPase upon SidK binding. Removal of the C-terminal residues 415–573 of SidK did not significantly affect V-ATPase inhibition by the SidK mutants (Fig 3A), but truncations of the N-terminal region of the protein has been demonstrated to prevent V-ATPase inhibition [25]. These data show that binding of the N-terminal domain of SidK to the V-ATPase is responsible for its partial yet specific inhibition of V-ATPase activity. The role of the C-terminal domain of SidK *in vivo* remains unclear.

## Discussion

In the data presented here, we determined the structure of residues 16–278 of SidK by X-ray crystallography. We showed that the N-terminal α-helical bundle of the protein binds with sufficient affinity to the catalytic A-subunit of V-ATPase to determine a cryo-EM structure of the V-ATPase:SidK_3_ complex. The interaction between SidK and the ‘non-homologous’ region of the V-ATPase A-subunit explains the specificity of SidK to V-type rotary ATPases. However, we observed that binding of purified SidK to purified and detergent solubilized yeast V-ATPase *in vitro* leads to only a ~40% inhibition of V-ATPase activity. It is possible that SidK induces a larger inhibitory effect on the macrophage V-ATPase than on the yeast V-ATPase. However, sequence alignment (S4F Fig) shows that the A-subunit from both human and yeast V-ATPase are highly conserved in their binding site for SidK, suggesting that at least binding affinity is similar.

The partial inhibition of V-ATPase is consistent with the observed effects of SidK binding on V-ATPase structure and TNP-ATP binding affinity. Complete inhibition of purified V-ATPase by bafilomycin shows that the V_1_ region (where ATP hydrolysis occurs) and V_O_ region (where bafilomycin binds) are fully coupled in the preparation of the enzyme used in these studies. The 40% inhibition appears to contradict the previous observation that purified SidK is a potent inhibitor of ATPase activity in yeast membrane vesicles [25]. However, the current *in vitro* assays with purified components [40] are more precise than the assays used previously, and complete inhibition of V-ATPase by an intracellular pathogen would be unexpected as it would ultimately kill the host cell. The amount of SidK expressed and translocated into the host cell cytoplasm could affect how SidK influences the host cell but it seems unlikely that inhibition would ever exceed the 40% observed in the *in vitro* assay without compromising the utility of the infected cells for the pathogen. Early in infection, *L*. *pneumophila* maintains a neutral pH in the *Legionella* containing vacuole (LCV) [32]. This stage of infection corresponds to the period where SidK expression is high [25]. However, it was also found that *L*. *pneumophila* with SidK deleted is still infectious and did not exhibit a detectable growth defect in either mouse bone marrow-derived macrophages or *Dictyostelium discoideum* [25]. This observation supports the idea that, as with most *Legionella* effectors, there are multiple functionally-redundant effectors that serve to neutralize the pH of the LCV during the early stages of infection. This idea is also consistent with the amount of inhibition of V-ATPase caused by SidK, which likely functions along with other, currently unknown, factors to control the pH of the LCV. SidK is not expressed at late phase of *L*. *pneumophila* infection, which may allow V-ATPase to lower the pH in the LCV, a condition that appears to benefit intracellular bacterial replication [32]. Thus, the coordinated expression of multiple effector proteins that affect the pH of the LCV would provide *L*. *pneumophila* with a level of control over LCV pH that would not be possible with a single effector protein that is a potent V-ATPase inhibitor.

A recent report showed that SidK binds the V-ATPase with a K_d_ of ~3.5 nM [41]. This high affinity suggests that even translocated at a low level in the host cell, SidK will bind to available V-ATPase complexes and associate with the LCV, which is known to possess V-ATPase at its membrane [42]. V-ATPase has been proposed to interact with numerous other proteins in cells, including ARNO and Arf6 [7], actin [43], aldolase [44], and ragulator [11]. It is possible that SidK binding to V-ATPase alters one of these interactions leading to downstream consequences in addition to V-ATPase inhibition. Although SidK clearly causes partial inhibition of the V-ATPase upon binding, it does so without causing significant structural changes in its target. Numerous other proteins inhibit rotary ATPases, but their binding usually involves more dramatic alteration of rotary ATPase structure and function. Inhibitory Factor 1 (IF1) prevents ATP hydrolysis in the mitochondria F-type ATP synthase [45] and the ε-subunit of the bacterial F-type ATP synthase inhibits ATP hydrolysis in that enzyme [46]. However, unlike IF1 and the ε-subunit, SidK binds to an interface on the A-subunit that is far from the catalytic site of the enzyme. The SidK binding site also differs from that of PA1b from *Pisum sativum*, which inhibits insect V-ATPases by binding to the c-subunits [47]. It is tempting to speculate that the C-terminal regions of SidK, which extend toward the membrane in the V-ATPase:SidK_3_ structure, may play additional roles in infection. This hypothesis is particularly appealing because the majority of the intact SidK protein is not necessary for interaction with the V-ATPase and is accessible to serve some other function. The inability of SidK to cause significant structural changes in V-ATPase is consistent with the need for V-ATPase activity as the infection proceeds. Two of five point mutations tested were sufficient to prevent inhibition of V-ATPase by SidK. Three of the five mutations engineered to test the proposed SidK:V-ATPase interface did not alter SidK’s affect on yeast growth. For the G24E mutation, this null result is explained by reduced expression of the construct. Mutations Y28A and W122A appear to be simply insufficient to abrogate inhibition by SidK. Given their important role in the defense against intracellular pathogens, V-ATPases are likely modulated by other intracellular pathogens during infection. The hybrid structural analysis procedure described here is uniquely capable of understanding the formation of these host-pathogen protein complexes.

## Materials and methods

### Cloning of SidK for X-ray crystallography

Truncated constructs of SidK (Q5ZWW6_LEGPH) were designed and created taking into account the secondary structure and disorder predictions [48,49]. Two constructs of SidK were cloned for crystallization trials: the full-length protein (residues 2–573) and a truncated N-terminal domain (residues 16–278). The gene *Lpg0968* was PCR-amplified from the genomic DNA of *L*. *pneumophila* strain Philadelphia 1 using the following primers: forward (2–573) 5’- TACTTCCAATCCAATGCCTCTTTTATCAAGGTAGGTATAAAAATGGG -3’, reverse (2–573) 5’- TTATCCACTTCCAATGTTA AAGGCTTAGGCTTTCTTCCTGTACTTT-3’; forward (16–278) 5’- TACTTCCAATCCAATGCCGAGCAATATCATAGTCAAGTAGTCGGT -3’, reverse (16–278) 5’-TTATCCACTTCCAATGTTATTTGCTTAAAGCATTTAATTTTTCGTTTTC-3’. The PCR products were cloned by Ligation Independent Cloning (LIC) into the LIC vector pMCSG7 [50] with the standard protocol [50]. The pMCSG7 vector encodes an N-terminal 6×histidine tag, separated from the target gene by a TEV protease cleavage site. The constructs produced, pCX0016 (SidK2-573) and pCX0038 (SidK16-278), were verified by DNA sequencing.

### Expression and purification of SidK(16–278) for X-ray crystallography

Competent BL21(DE3) pLysS cells (EMD Millipore Corp., Billerica, MA, USA) were transformed with the expression vectors described above. Single colonies were used to inoculate LB media (50 mL, 100 μg/mL ampicillin) and were grown overnight at 37°C. 1L TB media (100 μg/mL ampicillin) were inoculated with 50 mL of the overnight cultures and grown at 37°C with shaking at 220 RPM. Isopropyl β-D-1-thiogalactopyranoside (IPTG) was added to 0.5 mM when the OD_600_ of the cultures reached 2.0, and cells were grown for another 16 h at 18°C before being harvested by centrifugation for 20 min at 5,000 g. SidK(16–278) labeled with selenomethionine was expressed in methionine auxotroph B834(DE3) (Novagen). For selenomethionine labeling cells were grown in 1 L M9 medium supplemented with methionine at 37°C. When the OD_600_ reached 1.0 cells were centrifuged and resuspended in 1 L of M9 medium and the culture was grown at 37°C for another 4 h to deplete remaining methionine. After depletion, 50 mg/L of selenomethionine was added to the cell culture 30 min prior to the addition of IPTG.

Cell pellets were resuspended in buffer A (50 mM Tris pH 7.8, 400 mM NaCl, 0.5 mM tris(2-carboxyethyl)phosphine (TCEP)) with 1 mM p-aminobenzamidine and lysed with a high-pressure cell disrupter TS series Benchtop (Constant Systems Ltd, UK) at 35 psi. After centrifugation for 30 min at 16,000 g, the supernatant was loaded onto 5 mL of TALON Metal Affinity Resin (Clontech Laboratories, Inc., USA), incubated for 2 h at 4°C, and washed with 20 resin volumes of buffer A. Protein was eluted with buffer A with 100 mM imidazole. Protein was then dialyzed against 2 L of buffer B (20 mM 4-(2-hydroxyethyl)-1-piperazineethanesulfonic acid (HEPES) pH 8.0, 150 mM NaCl, 0.5 mM TCEP), and cleaved with TEV protease (1:50 (w:w) ratio, 16 h at 4°C) to remove the 6×histidine tag. Protein was purified further by gel filtration with an ENrich SEC 70 10 x 300 column (Bio-Rad Laboratories Inc., USA) in buffer C (20 mM HEPES pH 8.0, 150 mM NaCl, 0.5 mM TCEP). The protein eluted in two peaks: ~ 10% as an apparent dimer and ~ 90% as a monomer. The dimer peak fractions were pooled and concentrated to 18 mg/mL for the crystallization screening. After initial unsuccessful crystallization trials, the monomer peak fractions were subjected to reductive methylation using the Reductive Alkylation Kit (Hampton Research, Aliso Viejo, CA, USA), purified again by gel filtration, and the peak fractions were concentrated to 30 mg/mL for crystallization screening. SidK mutations were made by site directed mutagenesis (New England Biolabs). Protein melting temperatures were measured with the fluorescent dye Sypro Orange (Molecular probes) using an Applied Biosystems StepOnePlus Real Time PCR Instrument (Life technologies) according to the standard TmTool protocol developed by the manufacturer.

### Crystallization and X-ray data collection

SidK(16–278) dimer fractions produced well-diffracting crystals during crystallization screening with a Crystal Gryphon robot (Art Robbins Instruments, USA) using a Hampton Research Crystal Screen. The best crystals grew at 20°C with the well solution containing 0.1 M sodium cacodylate trihydrate pH 6.5, 0.2 M magnesium acetate tetrahydrate, and 20% (w/v) polyethylene glycol 8,000. The best crystals of Se-Met derivatized SidK(16–278) dimer were obtained at 15°C by microseeding with well solution containing 0.1 M 2-(N-morpholino)ethanesulfonic acid (MES) pH 6.5, 0.2 M magnesium acetate tetrahydrate, 0.13 M succinic acid pH 7.0 (Hampton Research) and 18% (w/v) polyethylene glycol 8,000. The initial crystallization conditions for the methylated monomer of SidK(16–278) were also identified using the Hampton Research Crystal Screen and vapor diffusion method. The best crystals were obtained at 20°C with wells containing 0.1 M Tris hydrochloride pH 8.5, 0.2 M lithium sulfate monohydrate, and 30% (w/v) polyethylene glycol 4,000. For data collection, crystals were soaked in a cryo-protectant (reservoir solution supplemented with 15% glycerol) and flash-cooled in liquid nitrogen. The X-ray diffraction data were collected at 100 K on the 08ID-CMCF (SidK-N dimer) and CMCF-BM 08B1-1 [SidK(16–278) methylated monomer] beamlines at the Canadian Light Source (Saskatoon, SK, CA) [51]. Diffraction data from the Se-Met crystals were collected at the Se absorption edge wavelength of 0. 9788 Å. The native and Se-Met datasets were processed and scaled with XDS [52].

### Structure solution and refinement

The positions of heavy atoms were found with SHELXD [53] and the initial model of the SidK(16–278) dimer was built with Phenix AutoBuild [54]. The model was then refined against a higher-resolution native dataset using the PHENIX software package [54] combined with manual rebuilding using Coot [55]. The structure of the SidK(16–278) monomer was solved using the molecular replacement program Phaser [56]. Asp125-Pro235 of SidK(16–278), was used as the initial model. This portion of the molecule was fixed as a partial solution, and then the N-terminal fragment of SidK(16–124) was used as a search ensemble. The resulting model was refined with phenix.refine and manually rebuilt in Coot. The structures were validated with MolProbity [57]. The details of data collection and refinement statistics are given in Table 1. Search for structural homologs of SidK (16–278) was performed with the DALI server [58], PDBeFold [59] (http://www.ebi.ac.uk/msd-srv/ssm), and deconSTRUCT [60].

**Table 1:** Summary of data collection and refinement statistics. The information for the highest resolution shell is given in parentheses.

	SidK dimer (native)	SidK dimer (SeMet)*	SidK monomer (native)
Wavelength (Å)	0.9794	0.9788	1.7712
Resolution range (Å)	45.34–2.4 (2.46–2.40)	46.9–2.80 (2.87–2.80)	48.9–2.3(2.36–2.30)
Space group	C2	C2	P4_3_
Cell parameters a,b,c (Å), β(◦)	162.1, 41.1, 94.2, 108.4	204.4, 41.0, 94.2, 95.2	52.96, 52.96, 127.44
Total reflections	145997 (10857)	145851 (11043)	233089 (16696)
Unique reflections	23542 (1731)	37431 (2820)	30728 (2255)
Multiplicity	6.2 (6.3)	3.9 (3.9)	7.6 (7.4)
Completeness (%)	99.9 (99.8)	99.8 (100)	99.9(99.5)
Mean I/Sigma(I)	13.4 (1.98)	8.9 (1.58)	16.8 (2.18)
Wilson B-factor (Å^2^)	44.51	65.5	37.62
R_merge_	0.115(1.105)	0.114 (1.018)	0.096(1.063)
R_meas_	0.125 (1.114)	0.132 (1.179)	0.103(1.114)
CC1/2	0.998 (0.70)	0.997 (0.651)	0.999 (0.758)
R_work_	0.195		0.17
R_free_	0.23		0.21
Ramachandran plot			
Favoured (%)	98		99
Allowed (%)	2		1
Outliers (%)	0		0
Clash score	1.69		0.96
Rmsd			
Bonds (Å)	0.002		0.002
Angles (◦)	0.454		0.496
PDB code	5UF5		5UFK

* Friedel pairs counted as independent reflections.

### Protein purification for cryo-EM and fluorescence experiments

V-ATPase and V_1_ were purified from *S*. *cerevisiae* as described previously [23] via a C-terminal 3×FLAG tag on the A-subunit. The SidK gene was cloned into a pET28 plasmid containing an N-terminal 6×histidine tag followed by a tobacco etch virus (TEV) cleavage site. BL21 Codon+ cells containing the SidK plasmid were grown at 37°C with shaking (225 RPM) in 1–4 L of LB media supplemented with 0.4% (w/v) glucose and 50 mg/L kanamycin. At an OD_600_ of 0.7, protein expression was induced with 1 mM IPTG and cells were grown overnight at 16°C. All subsequent steps were performed at 4°C. Cells were harvested by centrifugation at 5,000 g and lysed by sonication in TBS (50 mM Tris-HCl pH 7.4, 0.3 M NaCl) containing 0.001% (w/v) phenylmethanesulfonylfluoride (PMSF). Cell lysate was centrifuged at 38,000 g and the supernatant was loaded onto a HisTrap Ni-NTA column (GE Healthcare). The HisTrap column was washed in TBS containing 25 mM imidazole and SidK was eluted in TBS containing 0.3 M imidazole. SidK was mixed with TEV protease and dialyzed overnight in 2 L of TBS buffer containing 1 mM dithiothreitol (DTT). Cleaved protein was dialyzed in 2×1 L of TBS and passed through a HisTrap column. The HisTrap column was washed with TBS containing 25 mM imidazole and the flow through and wash were collected. Fractions corresponding to SidK were pooled and exchanged into ion exchange buffer (50 mM Tris-HCl pH 7.4, 1 mM ethylenediaminetetraacetic acid (EDTA)) by concentration and dilution in a centrifuged concentrating device (EMD Millipore). SidK was loaded onto a HiTrap Q anion exchange column (GE Healthcare) and eluted with a gradient of 0 to 200 mM NaCl. Fractions corresponding to SidK were pooled and exchanged into TBS containing 5 mM DTT by concentration and dilution in a centrifuged concentrating device (EMD Millipore).

### V-ATPase:SidK complex preparation

To purify substoichiometric V-ATPase:SidK assemblies, SidK from after TEV-cleavage was added to detergent-solubilized yeast vacuolar membranes and the V-ATPase:SidK complex was purified as described previously [23] except with twice the amount of washing. To purify fully-bound V-ATPase:SidK_3_ assemblies, TEV-cleaved SidK purified from the second HisTrap column (see protein purification above) was added to detergent-solubilized yeast vacuolar membranes and the protein complex was purified as described above.

### ATPase activity assay

ATPase activity assays were performed as described previously [23] in assay buffer (50 mM Tris-HCl pH 8, 0.05% [w/v] n-dodecyl β-D-maltoside (DDM), 3 mM MgCl_2_, 1 mM DTT, 0.2 mM NADH, 10 U pyruvate kinase, 25 U L-lactic dehydrogenase, 1 mM phosphoenolpyruvate, 2 mM ATP). Proteins were incubated on ice until assaying. 160 μL reactions were performed at room temperature using a 96-well plate in a Spectramax M2 UV/visible light plate reader (Molecular Devices). Four replicate experiments were conducted for enzyme with SidK or enzyme with buffer using the same stock of freshly purified V-ATPase or frozen ATP synthase. The final concentrations of enzyme and SidK were ~1 nM and ~30 nM, respectively. The concentration of bafilomycin used was 6 μM. In order to assay multiple constructs of SidK simultaneously for S5 Fig, samples had to be frozen and thawed to coordinate experiments, which decreases the ability of SidK to inhibit V-ATPase.

### Yeast growth assay

Yeast strain BY4741 [61] was transformed by the standard lithium acetate method [62] with plasmid p425GPD [63] carrying wild-type SidK or SidK mutants. The resulting strains were grown to saturation overnight at 30°C in Leucine dropout minimal medium (pH 5.5) and diluted at 1:40 into either minimal medium at pH 5.5 or minimal medium buffered with 50 mM MES and 50 mM MOP to pH 7.0. The cultures were incubated with vigorous shaking for 24 hrs at 30°C and yeast growth was monitored spectrophotometrically at 600 nm. Yeast cell lysates for western blotting analysis were prepared as described previously [25].

### Fluorescence titration

Fluorescence emission spectra of 2',3'-O-(2,4,6-trinitrophenyl) adenosine 5'-triphosphate (TNP-ATP) from 485 nm to 600 nm (10 nm slit width) were recorded with a Quantamaster QM-80 spectrofluorometer (Photon Technology International) using an excitation wavelength of 465 nm (4 nm slit width). The temperature was maintained at 10.0°C with a Peltier unit. Samples (0.5 mL) contained 0.25 μM V_1_, with or without 2.5 μM SidK, and varying amounts of TNP-ATP in assay buffer (25 mM Tris-HCl, pH 7.9, 0.15 M NaCl, 1 mM MgCl_2_, and 0.5 mM DTT). For each titration reading, 20 μL of the reaction sample was removed and replaced with 10 μL of trinitrophenyl-ATP (TNP-ATP) at different concentrations and 10 μL of 0.5 μM V_1_ in 2× assay buffer with or without 5.0 μM SidK. Fluorescence curves were corrected by baseline subtraction using the curve for [TNP-ATP] = 0. No significant fluorescence from the protein was observed. The increase in fluorescence due to addition of TNP-ATP was modelled in GNUplot (www.gnuplot.info) with the *fit* command using the equation [64,65]

$$I=\frac{\left[ES\right]}{\left[E_{T}\right]}I_{b}+m\left(\left[S_{}\right]-\left[ES\right]\right)$$ (1)

Where *I* is the fluorescence intensity, *I*_*b*_ is the fluorescence intensity of total enzyme bound to substrate, [*E*_*T*_] is the total enzyme concentration, [*S*_*T*_] is the total substrate concentration, and *m* is the increase in fluorescence per unit increase in free substrate. [*ES*] is the concentration of enzyme bound to substrate given by:

$$\left[ES\right]=0.5\left\{\left[E_{T}]+[S_{T}\right]+K_{d}-\sqrt{{\left(\left[E_{T}]+[S_{T}\right]+K_{d}\right)}^{2}-4\left[E_{T}\right]\left[S_{T}\right]}\right\}$$ (2)

where *K*_*d*_ is the equilibrium dissociation constant given by *K*_*d*_ = [*E*][*S*]/[*ES*]. The *m* parameter was estimated from fitting a straight line through the points corresponding to the four highest [TNP-ATP] values in each titration dataset. Each titration dataset was then modeled with fixed *m* to calculate *I*_*b*_ and *K*_*d*._ The experimental setup described here is similar to the study by Kubala *et al*. [65]. However, the tight binding affinity between TNP-ATP and the V_1_ subcomplex allowed parameters to be estimated from the titration data instead of from separate experiments. This experimental approach is advantageous in situations where protein availability is limited, which was the case in this study. Five replicate experiments were performed for each condition (with and without SidK) using the same batch of freshly purified V_1_ subcomplex.

### Cryo-EM imaging and image analysis

3 μL of ~10 mg/mL V-ATPase:SidK sample was applied to nanofabricated holey carbon grids [66] previously glow discharged in air for 2 mins. Excess sample was blotted away and the grid was plunge-frozen in a liquid propane-ethane mixture using a modified Vitrobot Mark III grid preparation robot (FEI company). Vitrified samples were imaged with a FEI Tecnai TF20 electron microscope operating at 200 kV and 34,483× magnification, resulting in a pixel size of 1.45 Å/pixel. Images were collected on a Gatan K2 Summit direct detector device operating in counting mode. 15 s movies were recorded at 2 frames/s and 5 e^-^/pixel/s. Movie frames were aligned using *alignframes_lmbfgs* and averaged with *shiftframes* [67]. Contrast transfer function (CTF) parameters of the averaged images were measured with *CTFFIND3* [68] and corrected for magnification anisotropy using *star_fixmaganiso* [69]. Candidate particle image coordinates were identified automatically in averaged images with *TMaCS* [70] using templates that were 2D projections of an existing map of the V-ATPase that had been low-pass filtered to 20 Å [23]. Candidate particle images were extracted from the raw movies and corrected for local drift using *alignparts_lmbfgs* [67]. The aligned and averaged particle images were corrected for magnification anisotropy with *correctmaganisotropy_fspace_list* [69] and processed by 2D and 3D classification and 3D refinement in *Relion* [71]. To minimize the possible influence of SidK on the classification algorithm, image classification was performed with masked maps to focus classification on the V_1_ region.

Maps were visualized and segmented in *UCSF Chimera* [72]. Atomic models of the V-ATPase (PDB 3J9T, 3J9U, 3J9V) were docked rigidly into the density maps using *UCSF Chimera* and flexibly fit into the maps with *Molecular Dynamics Flexible Fitting* (MDFF) [73]. Flexible fitting in MDFF was performed with implicit solvent and using backbone atoms only while restraining secondary structure. The atomic model of SidK was docked into the V-ATPase:SidK_3_ map using *UCSF Chimera* by rigidly docking alpha-helical bundles I and II/III into the map as independent domains. The domains were joined in *UCSF Chimera* and the geometry of the short connecting loop between the domains was optimized using Modeller [74]. The secondary structure of SidK was predicted using the online server *JPred* [75].

### Statistics

Where appropriate, results were analyzed using an unpaired, two-tailed Student's t-test (*TTEST* function in Microsoft Excel 2007) to calculate a p-value. Values less than 0.05 were deemed statistically significant.

## Supporting information

S1 Fig V-ATPase:SidK_3_ purification and cryo-EM. **A**, Sodium dodecyl sulfate polyacrylamide gel electrophoresis (SDS-PAGE) analysis shows co-purification of untagged SidK with intact V-ATPase (*left*) and the V_1_ subcomplex (*right*) via 3×FLAG tags on the V-ATPase A-subunits. **B**, Micrograph of V-ATPase:SidK_3_ complexes embedded in vitrified buffer. Examples of V-ATPase:SidK assemblies are indicated by red boxes. Scale bar: 30 nm. **C**, Trajectories of individual image features from the micrograph in **B** calculated using *alignparts_lmbfgs* [67]. **D**, Representative 2D class averages of V-ATPase:SidK_3_ complexes from 2D classification. Scale bars: 50 Å.
(TIF)

S2 Fig Image processing workflow and 3D classification scheme. Computational processing for images of fully-bound (**A**) and substoichiometric (**B**) V-ATPase:SidK complexes. Three distinct rotational states of V-ATPase were identified (states 1–3). The numbers in brackets (x:y:z) correspond to the average density of SidK relative to the V-ATPase A-subunit in the ‘tight’ conformation. The average density for SidK was determined by masking the SidK region and calculating the average value of the voxels in that region of the 3D density map. The numbers x, y, and z denote the density of SidK bound to A_1_, A_2_, and A_3_, respectively, where the V-ATPase A-subunit density is 100 and background is 0. Some classes show a mix of complexes with different SidK stoichiometries. **C**, Further classification allowed separation of substoichiometric V-ATPase:SidK complexes with different SidK binding configurations. Scale bar: 25 Å.
(TIF)

S3 Fig Cryo-EM maps of V-ATPase:SidK and assessment of resolution by Fourier shell correlation. **A**, Cryo-EM maps of V-ATPase:SidK complexes with angular distributions of image orientations. Longer red bars represent a larger number of images while shorter blue bars represent a smaller number of images. Scale bars: 25 Å. **B**, Fourier shell correlation (FSC) curves for masked maps constructed from gold standard refinement. Based on the 0.143 FSC cutoff, the overall resolutions of the ‘fully-bound’ maps for states 1, 2, and 3 are 6.8, 7.9, and 7.6 Å, respectively, and the ‘substoichiometric’ maps for states 1, 2, and 3 are 6.8, 7.7, and 7.7 Å, respectively. Resolutions were the same for FSCs with and without correction for masking in *Relion* [76].
(TIF)

S4 Fig Local resolution, particle image distributions, and comparison of V-ATPase structures with and without SidK bound. **A**, Local resolution estimates for maps of V-ATPase and V-ATPase:SidK. The poorly-resolved C-terminal region of SidK has a low resolution estimate. Similarly, the C-terminal region of the A-subunit in the ‘open’ conformation (A_open_) is at lower resolution in the V-ATPase map than in the V-ATPase:SidK_3_ map, indicating flexibility in this region. Scale bars: 25 Å. **B**, Classification of images with a mask around the V_1_ region shows that SidK binding increases and decreases the proportion of V-ATPase complexes adopting states 1 and 2, respectively. Three separate datasets were processed independently. Error bars represent one standard deviation. **C-E**, Overlay of V-ATPase models fitted into density maps of V-ATPase (*blue*) and V-ATPase:SidK_3_ (*pink*). Density of the a-subunit is shown as the transparent gray surface. No significant conformational changes in the V-ATPase were observed. Scale bars: 25 Å. **F**, protein sequence alignment of *S*. *cerevisiae* V-ATPase A-subunit with *Homo sapiens* V-ATPase A-subunit. Only the regions involved in the interaction with SidK are shown. Identical residues are shown in red.
(TIF)

S5 Fig The SidK mutants F62A and S85E prevent V-ATPase binding and inhibition. **A**, Point mutations F62A and S85E in SidK prevent the protein from co-purifying with detergent-solubilized V-ATPase. **B**, Point mutations F62A and S85E in SidK prevent the protein from inhibiting detergent-solubilized V-ATPase. *, p<0.01; **, p<0.001.
(TIF)

S6 Fig Differences in the catalytic region of the V-ATPase:SidK_3_ and V-ATPase density maps. The map of V-ATPase was subtracted from the map of V-ATPase:SidK_3_ and the difference map was low-pass filtered to 20 Å with a applied B-factor of 1000 Å^2^. Slices across the long axis of the structure were averaged for the N-terminal domains of the catalytic region (top row of images) and for the C-terminal domains of the catalytic region (bottom row of images). Residual density is observed in the difference map for the region corresponding to the A-subunit in the 'open' conformation (A_open_, arrows), indicating higher density in this region for the V-ATPase:SidK_3_ map compared to the V-ATPase map. Scale bars, 25 Å.
(TIF)
