# Supplementary figures and images for: Molecular basis for the binding and modulation of V-ATPase by a bacterial effector protein
Source: PLoS Pathog. 2017 Jun 1;13(6):e1006394. doi: 10.1371/journal.ppat.1006394 (PMC5469503; doi:10.1371/journal.ppat.1006394)

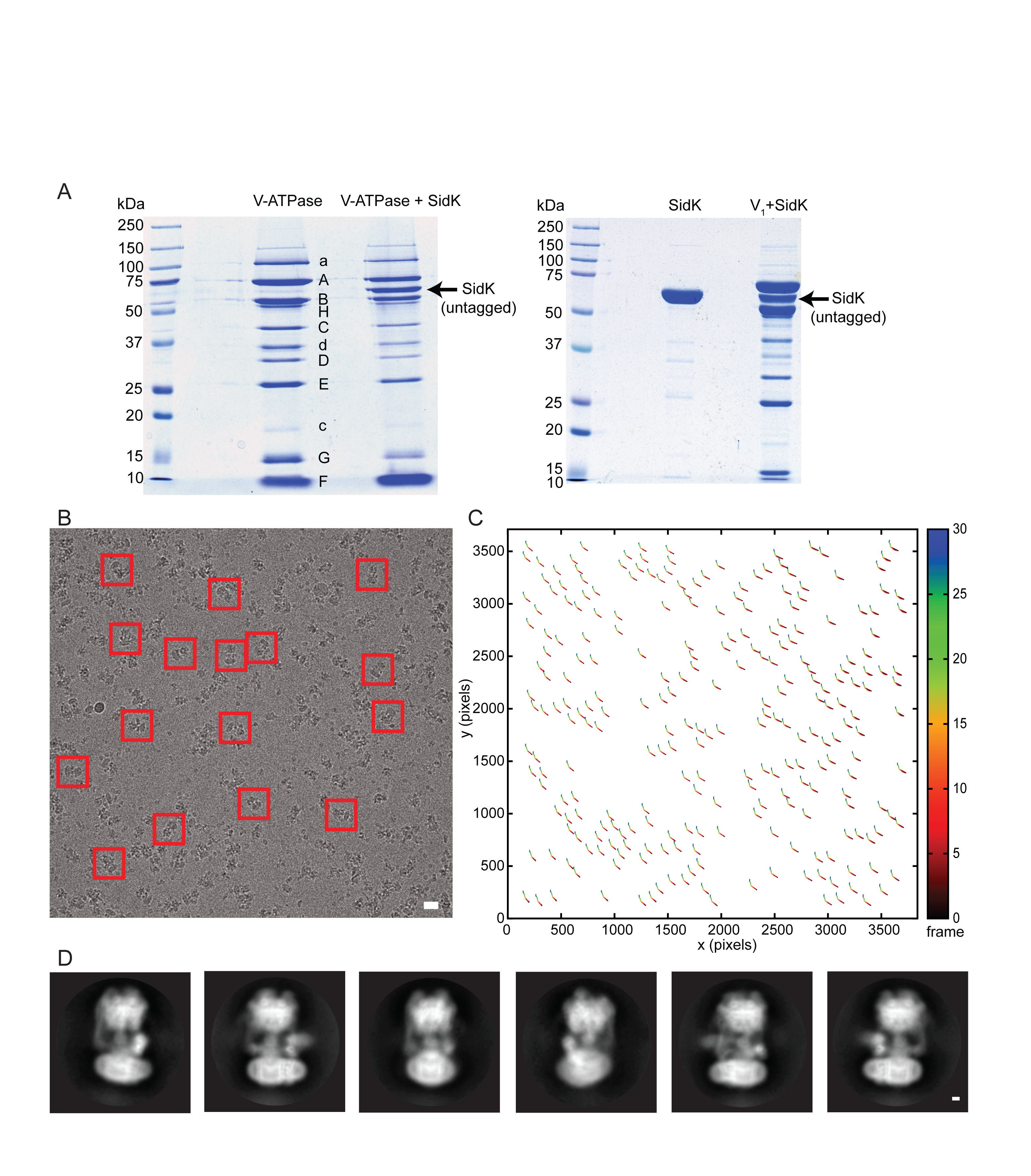

Supplement: S1 Fig — A, Sodium dodecyl sulfate polyacrylamide gel electrophoresis (SDS-PAGE) analysis shows co-purification of untagged SidK with intact V-ATPase (left) and the V1 subcomplex (right) via 3×FLAG tags on the V-ATPase A-subunits. B, Micrograph of V-ATPase:SidK3 complexes embedded in vitrified buffer. Examples of V-ATPase:SidK assemblies are indicated by red boxes. Scale bar: 30 nm. C, Trajectories of individual image features from the micrograph in B calculated using alignparts_lmbfgs [67]. D, Representative 2D class averages of V-ATPase:SidK3 complexes from 2D classification. Scale bars: 50 Å. (TIF) [file ppat.1006394.s001.tif]

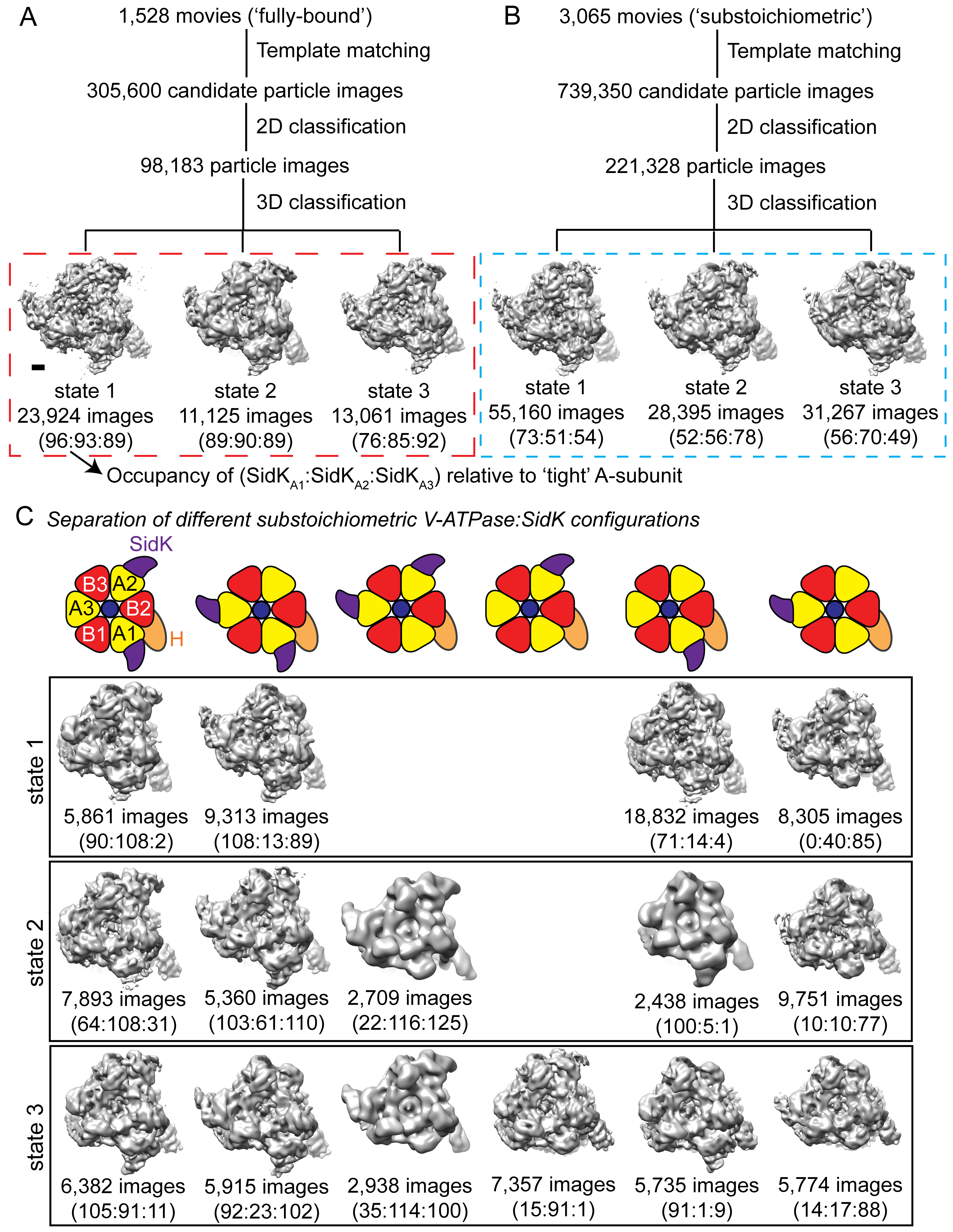

Supplement: S2 Fig — Computational processing for images of fully-bound (A) and substoichiometric (B) V-ATPase:SidK complexes. Three distinct rotational states of V-ATPase were identified (states 1–3). The numbers in brackets (x:y:z) correspond to the average density of SidK relative to the V-ATPase A-subunit in the ‘tight’ conformation. The average density for SidK was determined by masking the SidK region and calculating the average value of the voxels in that region of the 3D density map. The numbers x, y, and z denote the density of SidK bound to A1, A2, and A3, respectively, where the V-ATPase A-subunit density is 100 and background is 0. Some classes show a mix of complexes with different SidK stoichiometries. C, Further classification allowed separation of substoichiometric V-ATPase:SidK complexes with different SidK binding configurations. Scale bar: 25 Å. (TIF) [file ppat.1006394.s002.tif]

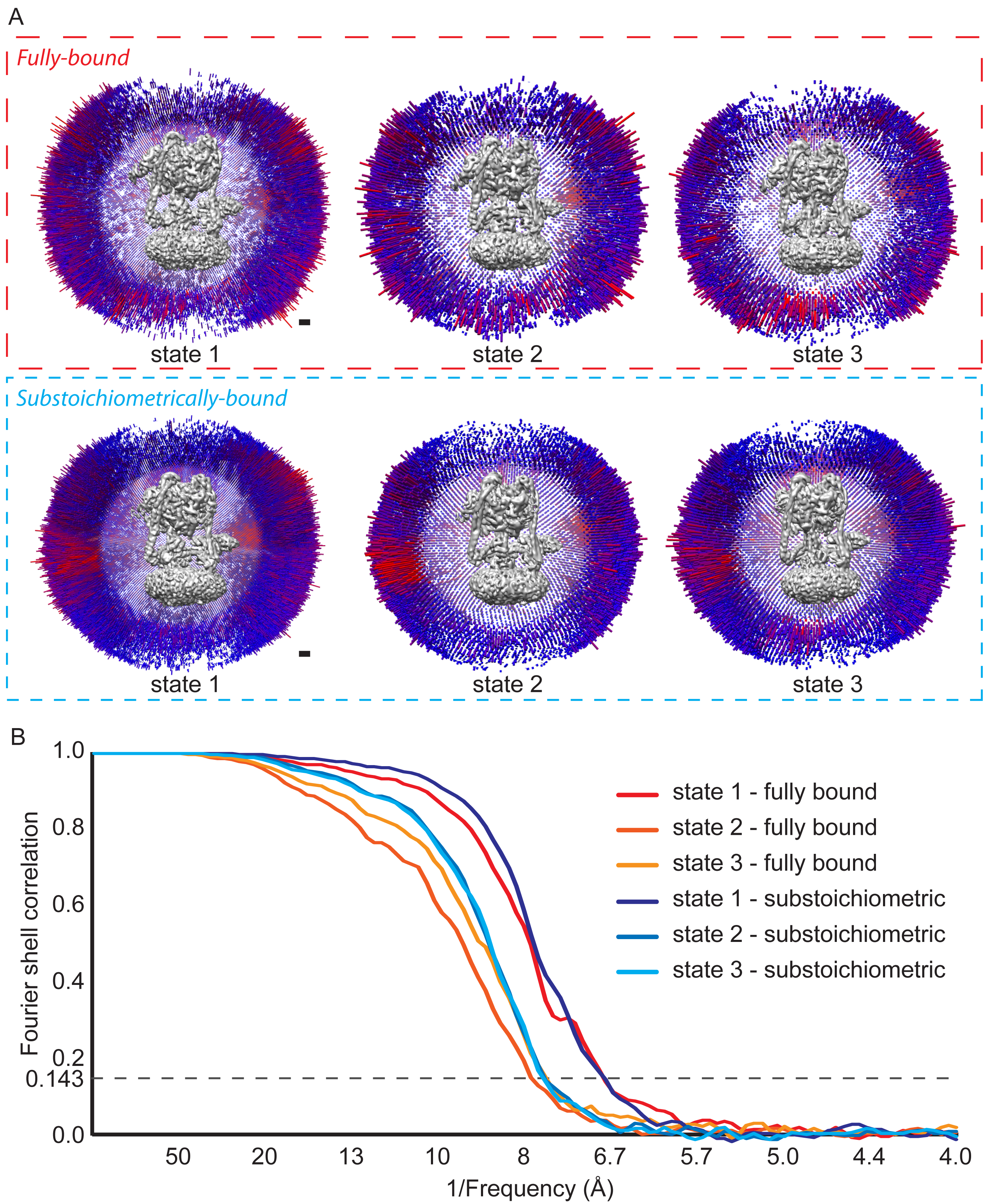

Supplement: S3 Fig — A, Cryo-EM maps of V-ATPase:SidK complexes with angular distributions of image orientations. Longer red bars represent a larger number of images while shorter blue bars represent a smaller number of images. Scale bars: 25 Å. B, Fourier shell correlation (FSC) curves for masked maps constructed from gold standard refinement. Based on the 0.143 FSC cutoff, the overall resolutions of the ‘fully-bound’ maps for states 1, 2, and 3 are 6.8, 7.9, and 7.6 Å, respectively, and the ‘substoichiometric’ maps for states 1, 2, and 3 are 6.8, 7.7, and 7.7 Å, respectively. Resolutions were the same for FSCs with and without correction for masking in Relion [76]. (TIF) [file ppat.1006394.s003.tif]

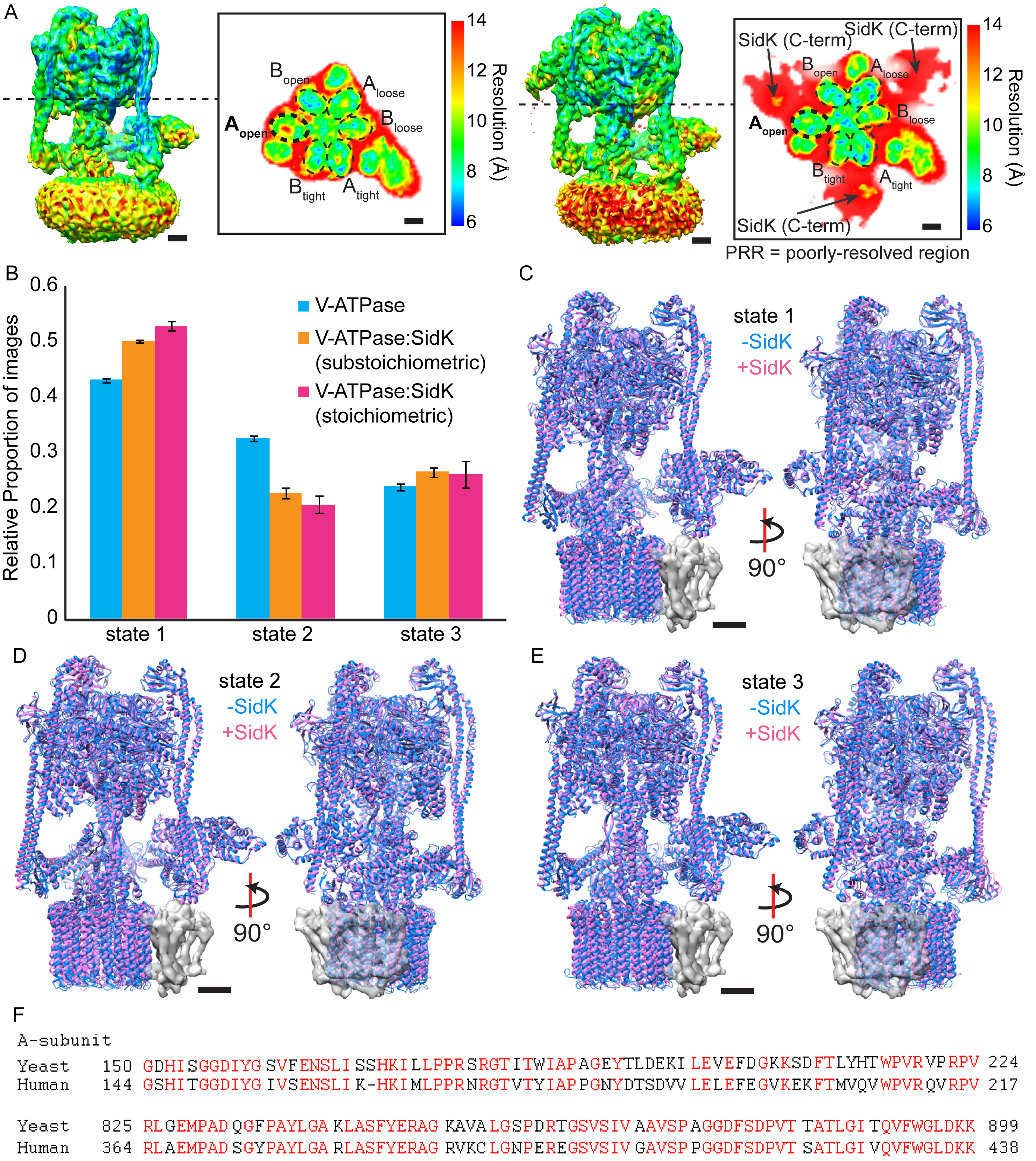

Supplement: S4 Fig — A, Local resolution estimates for maps of V-ATPase and V-ATPase:SidK. The poorly-resolved C-terminal region of SidK has a low resolution estimate. Similarly, the C-terminal region of the A-subunit in the ‘open’ conformation (Aopen) is at lower resolution in the V-ATPase map than in the V-ATPase:SidK3 map, indicating flexibility in this region. Scale bars: 25 Å. B, Classification of images with a mask around the V1 region shows that SidK binding increases and decreases the proportion of V-ATPase complexes adopting states 1 and 2, respectively. Three separate datasets were processed independently. Error bars represent one standard deviation. C-E, Overlay of V-ATPase models fitted into density maps of V-ATPase (blue) and V-ATPase:SidK3 (pink). Density of the a-subunit is shown as the transparent gray surface. No significant conformational changes in the V-ATPase were observed. Scale bars: 25 Å. F, protein sequence alignment of S. cerevisiae V-ATPase A-subunit with Homo sapiens V-ATPase A-subunit. Only the regions involved in the interaction with SidK are shown. Identical residues are shown in red. (TIF) [file ppat.1006394.s004.tif]

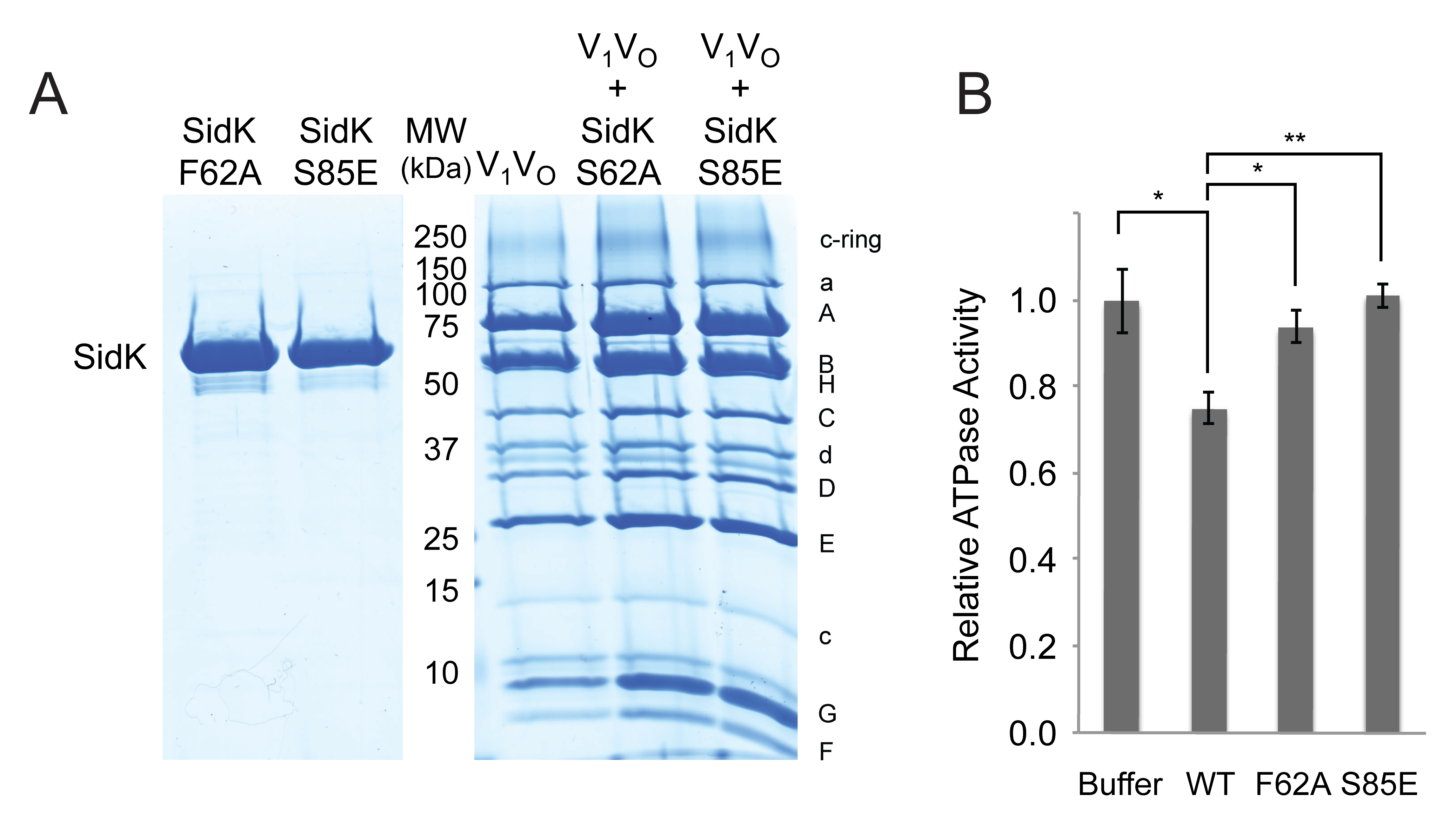

Supplement: S5 Fig — A, Point mutations F62A and S85E in SidK prevent the protein from co-purifying with detergent-solubilized V-ATPase. B, Point mutations F62A and S85E in SidK prevent the protein from inhibiting detergent-solubilized V-ATPase. *, p<0.01; **, p<0.001. (TIF) [file ppat.1006394.s005.tif]

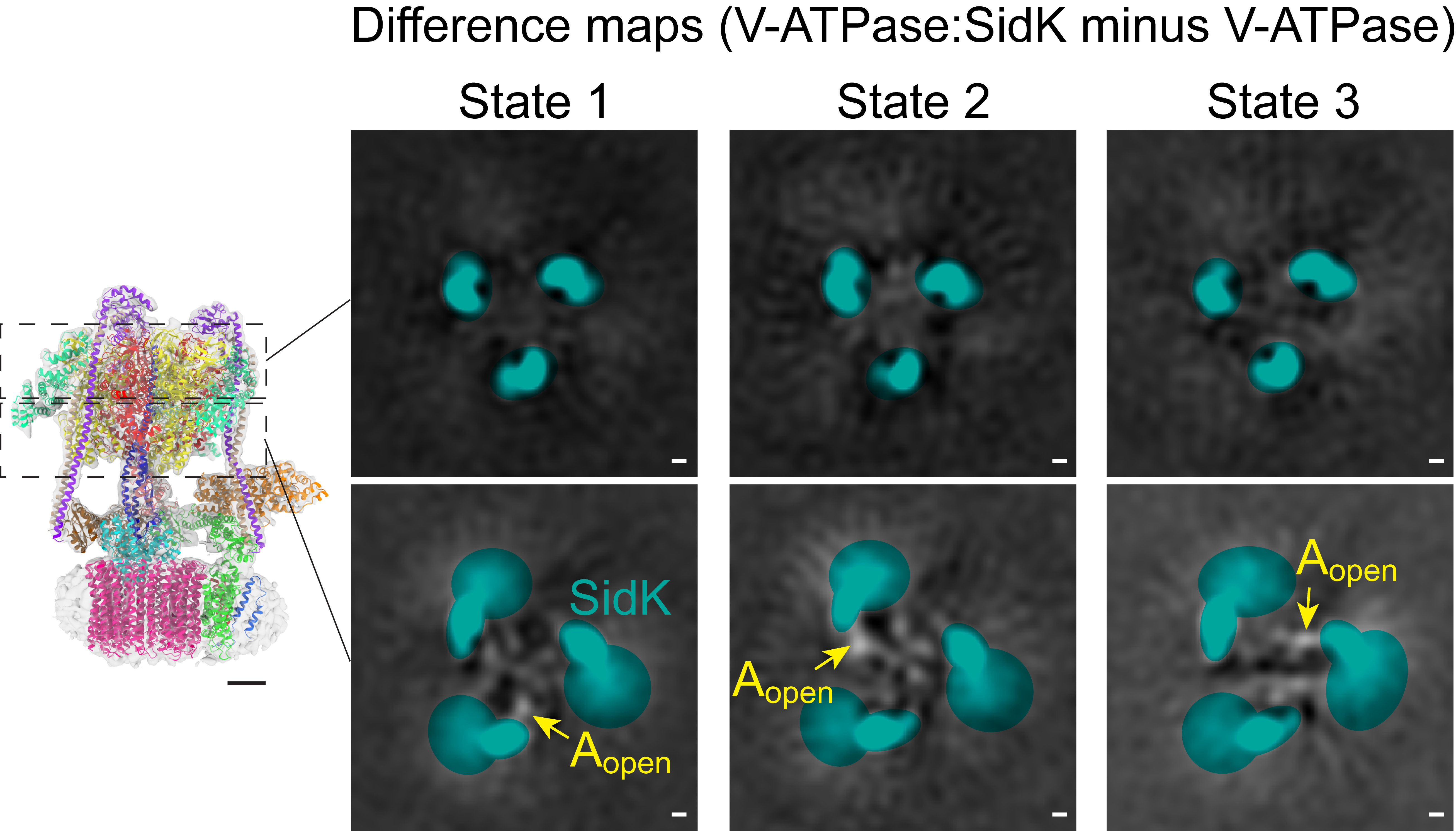

Supplement: S6 Fig — The map of V-ATPase was subtracted from the map of V-ATPase:SidK3 and the difference map was low-pass filtered to 20 Å with a applied B-factor of 1000 Å2. Slices across the long axis of the structure were averaged for the N-terminal domains of the catalytic region (top row of images) and for the C-terminal domains of the catalytic region (bottom row of images). Residual density is observed in the difference map for the region corresponding to the A-subunit in the 'open' conformation (Aopen, arrows), indicating higher density in this region for the V-ATPase:SidK3 map compared to the V-ATPase map. Scale bars, 25 Å. (TIF) [file ppat.1006394.s006.tif]
